# Latin America and the Caribbean: Assessment of the Advances in Public Health for the Achievement of the Millennium Development Goals

**DOI:** 10.3390/ijerph7052238

**Published:** 2010-05-06

**Authors:** Amal K. Mitra, Gisela Rodriguez-Fernandez

**Affiliations:** Department of Community Health Sciences, The University of Southern Mississippi, Hattiesburg, MS 39406, USA; E-Mail: guisito8@gmail.com

**Keywords:** Latin America and Caribbean, public health, Millennium Development Goal, global burden of disease, inequality

## Abstract

To improve health and economy of the world population, the United Nations has set up eight international goals, known as Millennium Development Goals (MDGs), that 192 United Nations member states and at least 23 international organizations have agreed to achieve by the year 2015. The goals include: (1) eradicating extreme poverty and hunger; (2) achieving universal primary education; (3) promoting gender equality; (4) reducing child mortality; (5) improving maternal health; (6) combating HIV/AIDS, malaria and other diseases; (7) ensuring environmental sustainability; and (8) developing a global partnership for development. Having been in the midway from the 2015 deadline, the UN Secretary-General urges countries to engage constructively to review progress towards the MDGs. This paper aims to evaluate advances in public health, with special reference to gender inequalities in health, health sector reform, global burden of disease, neglected tropical diseases, vaccination, antibiotic use, sanitation and safe water, nutrition, tobacco and alcohol use, indicators of health, and disease prevention in Latin America and the Caribbean region (LAC). The paper also identifies areas of deficits for the achievement of MDGs in LAC.

## 1. Introduction

The United Nations member countries and 23 international organizations have set up eight goals, known as Millennium Development Goals (MDGs), as a framework to assess current and future health trends around the world and to improve human development [1,2]. Although all of the eight goals stated by the MDGs influence health status of the people, at least four of them may be considered as indicators of public health of a nation; they include: promoting gender equality; reducing child mortality; improving maternal health; and combating HIV/AIDS, malaria and other diseases [1].

According to the Pan American Health Organization (PAHO), LAC region faced an important demographic transition, with a significant decline of mortality and fertility rates, and an increase of its population from 331 million in 1950 to more than 823 million in 1999, which represents almost 14% of the world’s population [3]. Moreover, while LAC region has made magnificent efforts in reducing mortality, especially among children, and in increasing life expectancy, little is known about the disparities between and within countries of the LAC region [3].

Because it is necessary to keep track of advances toward MDGs, this paper will focus on recent advances and challenges in public health in the LAC region. This manuscript is a review paper of health indicators that demonstrate the global burden of disease in the LACs in comparison with the United Nation’s MDG attainment. While the review will focus on the region as a whole, whenever necessary and data are available, some specific details will be offered for three countries: Bolivia, Brazil and Cuba. The logic is to present a comparison within the region among the poorest (Bolivia) and the richest (Brazil) countries, and to highlight the unique case of Cuba based on its health indicators.

## 2. Results and Discussion

### 2.1. Inequalities

Bolivia is a country located in the middle of South America, and according to the World Bank, it is the poorest country in the whole region [4]. Bolivia’s population size is 9 million, of which the most (64%) live in urban areas. According to the Human Development Report by the United Nations, Bolivia ranked 117th in the Human Development Indicator [5]. Moreover, a study conducted on health disparities among indigenous and non-indigenous Bolivians found that infant mortality in 139 of 327 rural municipalities was twice as high as in the region as a whole [6].

Brazil, on the other hand, is the fifth largest country in the world when referring to the population size [7]. Brazil is a country with one of the most uneven wealth distributions. Eighty-five percent of the nation’s wealth is controlled by only 5% of the population. Brazil’s economy is paradoxical. Even though it is classified as a developing country, it has the tenth largest economy in the world [8].

Cuba represents a paradox in many areas, including health indicators. Ever since the fall of the Soviet Union and the embargo imposed by the US, Cuba has been characterized by a profound economic crisis. According to PAHO, the country’s gross domestic product grew modestly at an annual rate of 4.8% from 1994 to 2000 [9]. It is remarkable that in spite of the economic recession of the early 1990s, Cuba continues to rank among the top five percent of 125 developing countries on indicators of social and health development such as life expectancy, infant and maternal mortalities, adult literacy, primary and secondary school enrolments and many others [10]. Studies showed that the economic crisis led Cubans eat less calories. The lack of public transport in Cuba meant residents used other means of transport, such as walking or biking. Food scarcity and increased energy expenditure made Cubans thinner. Between 1997 and 2002, there was significant decline in death from diabetes (51 percent), heart disease (35 percent), stroke (20 percent) and all causes (18 percent) in Cuba [11].

### 2.2. Gender Inequalities of Health and Healthcare

An influential body of research on gender inequalities of health has developed since the late 1960s. In Brazil, the gender gap in life expectancy at birth is nearly eight years; however, women’s higher life expectancy is offset by their lower healthy life expectancy at almost all ages, particularly among lower socioeconomic groups [12]. In developing countries, health problems which pose the greatest hardship to women include: reproductive health problems, violence, occupational and environmental hazards, osteoporosis, and breast cancer [13]. Women make up 70% of the 1.2 billion people living in poverty; they are usually either without medical insurance or are underinsured. In some societies, men get preferential medical care and early hospitalization than women for a similar illness [14]. Strategies to eradicate gender inequalities in health must involve efforts to improve the status of women in the society.

Safe abortion is a simple, lifesaving health service. Nonetheless, every year nearly 70,000 women die unnecessarily from the complications of unsafe and illegal abortions. The WHO estimates that 20 million of the 42 million abortions performed annually are illegal and unsafe. Every year four million women in Latin America have an illegal abortion [15]. In most LAC countries, a woman wishing to terminate a pregnancy has either to go abroad or risk an operation performed by unqualified medical personnel. As long as abortions remain illegal women will continue to die from illegally performed abortions. Safe abortion care is part of comprehensive reproductive health services, which should be accessible to and affordable for all women, as part of their reproductive rights.

### 2.3. Limitations of Health Sector Reform

Health sector reform (HSR) is one of the central issues in health services. Experience gathered from most LAC countries suggests that the implementation of HSR has not achieved the effects expected. In Colombia, even though there have been some achievements in reducing inequities in access and resource allocation, the health expenditures have increased greatly, which makes the extension of the benefits to the other half of the poor population who encounter themselves outside the system, unsustainable and unrealistic [16]. In Chile and Argentina, the reforms have created two segmented subsystems of medical insurance: public and private. The public system covers low income people with high health risks while the private system covers people with the highest income and low health risk [17]. Contracting out to private providers for the delivery of health care services was another strategy to improve the efficiency and quality of health care services in LAC. However, this large-scale privatization has been associated with managerial problems, particularly in regards to the monitoring and supervision of contracts and service providers such as in Guatemala. Expansion of private insurance often has generated additional co-payments, which have increased out-of-pocket expenditures, thereby worsening access to needed services [18]. Furthermore, different levels of quality in service delivery among providers have also been identified, limiting the equity of services to all [16].

### 2.4. Population Distribution in LAC

Population distribution and population age structure are crucial determinants of social, economical and health related services. From 1950 to 2000, the LAC population increased from 175 million to more than 515 million, and in 2008, the estimated population was 653 million [19]. Part of the explosive population growth that LAC experienced in the 20th century was due to high birth and rapid declining death rates. After Africa, LAC region was one of the fastest growing regions, but this population explosion stopped around 1970 due to the political instability of the region. During the same period, LAC experienced a shift in populations from rural to urban settings, therefore giving route to the creation of mega cities such as Sao Paulo, Mexico City and Buenos Aires [20].

The general population of LAC is young, with a vast proportion of children being under age 15. The decline in infant mortality rate (IMR) during the 1930s created the region’s youthful age structure as well [20]. Now-a-days, Bolivia, El Salvador, Guatemala, Haiti, Honduras, Nicaragua, and Paraguay have a 35% of their population under age 15 [20]. Moreover, it is predicted that by 2050, the LAC population will change from a pyramid shape to a more rectangular one. A pyramidal shape of a population distribution shows a large base (many young children) and a pointed top (a very small number of old people). In such populations, the older people (over age 60) are typically less than 5% of the population, whereas children below the age of 15 can constitute around 40%. High fertility and high mortality rates attribute to the pyramidal age structure. As birth rates start to decline, the baby boom comes to an end, and together with a decreased mortality and increased life expectancy, the population structure gives a more rectangular shape. In the later, the elderly population rises from 5% to nearly 20% of the population. Chronic diseases disproportionately affect older adults and are associated with disability, diminished quality of life, and increased costs for health care and long-term care. The rapidly increasing number of older people and diversity of disease pattern has far-reaching implications on the MDGs in the future. For countries with aging population, more focus is needed in managing chronic and degenerative diseases and for long-term care. To understand the transition in age structure, it is important to mention the age dependency ratio. Dependency ratio is defined as the ratio of the dependent population (those under age 15 and those aged 65 or older) to the working-age population (ages 15 to 64) [21]. In LAC, the dependency ratio is expected to decline until 2030, creating therefore a “demographic dividend” and giving the transitional countries the opportunity for economic growth.

Population mobility, especially internationally, has also reshaped LAC population distribution. Most Central Americans and Mexicans immigrate towards the US, while many South Americans have immigrated to Europe. The region has also experienced internal immigration, especially people from Bolivia, Chile, Paraguay and Uruguay moving into Buenos Aires and Brazil, and a vast majority moving into Venezuela [20]. Considering recent immigration waves from and to LAC, it is also crucial to take into consideration the overall health status of immigrants and their families.

### 2.5. Global Burden of Disease

Not so long ago, the dichotomy between communicable infectious diseases (CID) and non-communicable chronic diseases (CD) was believed to be well defined and marked. CIDs were more prevalent in developing countries, while CDs were more prevalent in developed countries. In today’s world this is not the case scenario anymore, and developed and developing nations are experiencing a phenomenon called epidemiological transition and a double burden of diseases [22]. The global burden of disease (GBD) 2030 projections take into account demographic predictors such as age, gender, and geographical region [22]. The GBD classifies diseases and health related conditions into three broad groups: group I diseases are communicable diseases that include maternal, perinatal and nutritional conditions; group II diseases are non-communicable diseases; and group III diseases are non-fatal health conditions such as injuries (Table 1). Based on estimates of 2002, there were 57 million deaths, and 10.5 of these deaths were children under age 5 [22]. Most of these deaths were from countries with low or middle income. A major finding was the increase of HIV/AIDS morbidity and mortality in the past decade. In 1990, HIV/AIDS accounted for only 2% of deaths corresponding to group I, whereas in 2002, it accounted for 14% of deaths worldwide, and it is estimated that by 2030, it will be among the top three causes of deaths around the world [22]. Mortality due to group I diseases are more common among infants and children under age 5. Moreover, 60% of all deaths in children are attributed to preventable conditions such as malaria, diarrhea, tetanus and pneumonia among others. Surprisingly, group II diseases account for more than 50% death in developing nations among adults of ages 15–59 years, where ischemic heart disease and stroke are dominant causes of death globally. This suggests that the epidemiological transition in developing countries is already taking place and must be considered for further health recommendations.

LAC countries, like the rest of the world, are also experiencing the GBD and the epidemiological transition. However, this transition has dramatic implications since now LAC not only is struggling with CID such as malaria and tuberculosis, but also with CD such as cardiovascular disease, malignant neoplasm, chronic obstructive lung disease and diabetes mellitus (Table 1).

**Figure 1.**
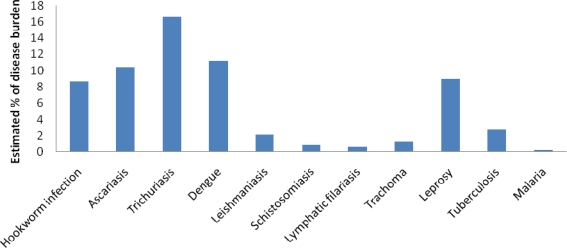
Neglected tropical diseases in the Latin American and the Caribbean Region, 2008. Source: modified from Hotez *et al.*, 2008 [24].

According to a PAHO strategic plan for 2008–2012, the average life expectancy in LAC region has increased to 74.6 years in 2005 [23]. However, the same document declared that the mortality and morbidity profile of the region is changing, placing CD as the leading causes of death along with communicable diseases [23]. On the other hand, during 2000–2004 there were approximately 58 deaths per 100,000 attributed to CID, which means that they are still major burden in this region [23]. Haiti, for example, is facing the highest incidence of tuberculosis and multiple drug-resistant tuberculosis in the region. In 2006, Brazil alone had 50% of dengue cases in the region [23]. Because most developed countries had achieved a great epidemiologic transition from CID to CD, attention has shifted into prevention of CD. The burden of neglected tropical and infectious diseases is, therefore, a challenge in many regions of the world, such as LAC. Figure 1 shows estimated percentage of disease burden of neglected tropical diseases in LAC.

Adding to the problem, CDs are responsible for approximately 60% of all deaths in the region, and the leading causes of CD are hypertension, obesity, hyperglycemia, and hyperlipidemia [23]. Moreover, while ischemic heart disease is the second leading cause of death around the world, its rate in LAC region is forecasted to double in the next years, along with stroke and diabetes [23]. Deaths from lung, breast and prostate cancer are also on the rise.

### 2.6. Vaccination

One of the greatest achievements of public health has been the development of vaccines and their crucial role in the eradication of infectious diseases including polio, tetanus, diphtheria, whooping cough, measles and many others [25]. PAHO launched a 1990–2004 strategy for immunization, which aimed to eliminate rubella; progress towards eliminating measles and polio; have a well equipped information management system; and introduce new and underutilized vaccines [26]. To illustrate, Bolivia, under the surveillance of PAHO, established an Extended Immunization Program (Programa Ampliado de Inmunizaciones) in 1979. In that year, general vaccination of children less than one year did not even reach 20% [27]. Ever since then, the program has succeeded in achieving its goal to involve the government and its communities in disease prevention programs [27].

The WHO immunization profile shows that Bolivia has experienced a decrease in reported cases of diphtheria, from 2 in 2004 to 0 in 2008. Moreover, no cases have been reported for measles, polio, and rubella from 2004 to 2008 [28]. Pertussis cases decreased from 6 in 2004 to 0 in 2008. Similarly, rubella had dramatically decreased from 12 cases in 2004 to 0 cases in 2008. Tetanus and yellow fever cases followed a similar pattern from 4 in 2004 to 0 in 2008, and 13 cases in 2004 to 1 in 2008, respectively [28].

From 2004 to 2008, Brazil’s immunization profile shows a dramatic decrease in diseases. No cases of measles and polio were reported during this period, except a peak of 57 cases of measles reported in 2006. Tetanus decreased from 463 cases in 2004 to 333 cases in 2008, and yellow fever decreased from 85 cases in 2004 to 46 in 2008. Conversely, from 2004–2008 there has been an increase of reported cases for the following diseases: diphtheria from 15 to 85 cases; pertussis from 1,146 to 3,562, and rubella from 16 to 30 [29].

Cuba stands for the total absence of reported cases from 2004 to 2008 on the following diseases: diphtheria, measles, pertussis, polio, rubella, and yellow fever. The only incidence of cases of vaccine-preventable diseases was for mumps, which decreased from 279 cases in 2004 to 11 cases in 2008, and tetanus, which presented 3 cases in both 2006 and 2008.

### 2.7. Antibiotic Use

Ever since the discovery of the first antibiotic by Alexander Fleming in 1927, antibiotics have transformed the natural history of diseases and have drastically reduced deaths and illnesses from infectious diseases [30]. Conversely, antibiotic resistance due to misuse and overuse of this has been called a major public health concern [30]. In LAC region antibiotic resistance is a great concern. As mentioned by Mainus, in LAC the use of antimicrobials without doctor’s prescription is widely due to the lack of laws and restrictions on antibiotic sales [31]. Moreover, health beliefs about antibiotics make its use even more indiscriminate. Due to a lack of control of people’s self-prescription and unrestricted use of antibiotics in most developing countries including Latin America, bacterial resistance to antimicrobial agents are increasing as one of most jeopardizes to global public health. Selective pressure due to wide availability of the antibiotic, improper dosage of the antibiotic, the quality of the antibiotic, and environmental chemical pollution (for example, ozone) are considered possible causes of the emergence and spread of multiple drug resistance [32]. In the last 10 years, many LAC governments, with the support of international organizations, have made remarkable efforts to regulate and control the proper use of antibiotics. Part of this effort is the creation of the Alliance for the Prudent Use of Antibiotics (APUA) in Cuba, Brazil and many other LAC countries. APUA-Cuba was established in 1996 and its current interests include surveillance programs to control and monitor drug resistance [33]. APUA-Brazil was created in 2001 and its current mission highlights the importance to promote the appropriate use of antibiotics and set forth the control of antimicrobial resistance through research and education activities [34]. Based on the APUA, Bolivia is not included among its affiliated chapters. However, Bolivia’s health minister declared that auto medication in this country exceeds 17% of its use, which results in antibiotic resistance [35]. An alternative response to this major issue in Bolivia and other LAC countries that have no APUA chapter is the creation of the South American Infectious Disease Initiative (SAIDI), a sub-regional strategy for the Andean countries with the general purpose to better contain the emergence and spread of antimicrobial resistance [36]. Moreover, since 2004 many studies supported by World Health Organization emphasize the importance to strengthen legislations on drug prescriptions, especially of antibiotics, and to weigh the influence and repercussions of judicial measures on the inappropriate use of antibiotics [37].

### 2.8. Sanitation and Safe Water

According to a survey conducted among 11,300 readers of the British Medical Journal, sanitation has been identified as the most important medical advance since 1840 [38]. In developing countries, sanitation has a strong and significant impact on the overall health of people. Today, almost 2.5 billion of people around the world lack proper sanitation facilities, and another 884 million do not have access to drinking water resources [39]. One major response comes from UNICEF approach to improve water supplies to increase sanitation facilities and to promote hygienic practices in more than 90 countries [39]. Besides, the WHO/UNICEF Joint Monitoring Program (JMP) for water supply and sanitation declared sanitation as its central focus in 2008 [40]. Even though there is still a serious need to increase access to sanitation and safe water, people’s awareness of the importance of sanitation has considerably increased [38]. Besides, the JMP shows that in the developing world, sanitation coverage has increased from 41% in 1990 to 53% in 2006 [40].

When compared to Southern Asia and sub-Saharan Africa, LAC region has committed and has received increasing support to scale the “sanitation ladder”. According to JMP, based on the global progress toward the MDG sanitation target, in LAC region the sanitation coverage for 1990 was 68%, showing an increase of 11% with a total coverage of 79% for 2006, and a MDG target coverage of 84% which is a progress expected to be on track. However, at the regional level, while most LAC is on track to meet the MDG, Bolivia stands as one of the few LAC countries that are not on track [40]. More to the point, when considering urban and rural sanitation coverage, Brazil and Bolivia have less than 50% of its rural areas covered, while Cuba has more than 91% of its rural areas covered. The urban coverage in Bolivia is between 50% and 75%, in Brazil it is 76% to 90% and in Cuba it is more than 91% [40].

Access to drinking water is also another major issue. From 1990 to 2006, there has been an increase of 1.6 billion people using drinking water from a safe source. Moreover, the JMP report declares that the world is on track to meet the drinking water target [40]. LAC is among the top regions whose population uses an improved drinking water source such as water pipes on premises, with a coverage that increased from 67% in 1990 to 80% in 2006 [40]. JMP shows that the progress towards MDG drinking water target for LAC countries increased from 84% in 1990 to 92% in 2006. The progress is on track to achieve the MDG target of 92% by 2015.

Regional details show that Bolivia, Brazil and Cuba are on track to meet the MDG drinking water target. However, while drinking water coverage in Brazil and Cuba is greater than 91%, Bolivia’s coverage is 76% to 90% [34]. Unfortunately, the urban and rural disparities of drinking water use are extreme in LAC countries. For example, the coverage in rural areas is 50% to 75% in Bolivia and Brazil, whereas it is 76% to 90% in Cuba [40].

### 2.9. Nutrition

Nutrition is both an indicator and outcome of national development. In one hand, LAC faces the problem of under-nutrition among children younger than 2 years. On the other hand, over-nutrition is an increasing problem among the adult population. Chronic under-nutrition, which is measured by stunting (low height-for-age), has been recognized as the most prevalent form of under-nutrition in the region [41]. In addition, macronutrient deficiency plays a major role in under-nutrition, and its consequences are reflected in many cases of iron deficiency anemia, vitamin A deficiency, and iodine deficiency [41]. Because nutritional anemia is a key indicator of under-nutrition, many LAC countries fortify foods such as wheat, corn and margarine with a combination of vitamin B, iron, folate, niacin, riboflavin, thiamin, and vitamin A [41]. Vitamin A deficiency is the top cause of childhood blindness in developing nations, and subclinical vitamin A deficiencies have been identified in the countries of Bolivia and Northern Brazil [41]. However, it is hard to assess the real extend of this problem since recent data from these regions are nonexistent. All LAC countries have initiated programs of vitamin A supplementation. Among the top efforts are the incorporation of vitamin supplementation into immunizations, and supplementation of postpartum women [41]. Moreover, major efforts are being placed on strengthening the link between vitamin A supplementation, the expanded program of immunization, and the integrated management of childhood illness [41]. Concerning iodine deficiencies, all LAC countries that are at risk have implemented national iodization programs. Salt iodinization has covered about 90% of households in LAC.

Obesity has become an emerging epidemic in the LAC region. According to PAHO, the prevalence of overweight children was between 25% and 30% in 2002 [41]. Obesity, however, is unevenly distributed by gender and socioeconomic status. Women are at an increased risk of obesity, mainly because of their less involvement in physical exercise and sports activities than their male counterparts. Studies in Guatemala showed that a higher proportion of women are obese than men (24% *vs*. 11%). Physical activity levels recommended to prevent obesity ranged from 24% in urban men to 77% in agricultural rural men, whereas it was only 3% in urban women and 2.3% in rural women [42]. The same pattern was observed in time spent in moderate/vigorous activities. Nearly all agricultural-rural men spent ≥60 min/day in moderate/vigorous activities, compared to 63% of nonagricultural-rural men, 39% of urban men, 12% of rural women and 22% of urban women [42]. The data on gender differences in physical exercise and moderate/vigorous activities could be explained by differences in time use between women and men. In a study conducted in six countries such as Argentina, Nicaragua, South Africa, Tanzania, Korea and India showed that the mean time spent by women on unpaid care work is more than twice the mean time spent by men [43]. If all types of work are combined, women allocate more time to work than men—which means women have much less time for leisure, education and self-care. International, regional and national attentions now focus on nutritional balance. For example, nutrition experts are insisting the food industries to support “trans Fat Free Americas”, a call made by PAHO to phase out trans-fats from food products [44]. Regional programs in combating obesity include “Agita Sao Paulo” in Brazil, “Muevete Bogota” in Colombia, and the physical activity network of the Americas, supported by PAHO and CDC [44,45].

### 2.10. Tobacco Use

The global youth tobacco surveillance, 2000–2007 of CDC shows that cigarette smoking is significantly higher in LAC, Europe and West Pacific regions. In Mexico and Brazil, the two most populous countries of LAC, an estimated 250,000 people died from tobacco related diseases in 2005 [46].

The economical cost for tobacco addiction is gigantic, with an estimate of 15% of LAC public budget spent on tobacco related diseases. Conversely, the ones obtaining a high capital from this epidemic are tobacco industries which, according to a report by the Corporate Accountability International, are accused of interfering with the region’s goal to decrease tobacco consumption [46]. For example, tobacco companies in Guatemala were heavily involved in lobbing politicians and officials who were working on the Framework Convention on Tobacco Control (FCTC) ratifications in 2005 [47].

Recent studies also suggest that the number of women using tobacco is rising in some developing countries. Doskoch mentioned that Latin American women had the highest level of tobacco use in comparison to women of the Middle East and Africa [48]. The study surveyed women from five LAC countries, and concluded that in each of the five countries, at least a third of women had ever tried a cigarette; three-quarters of women in Argentina and Uruguay and three-fifths of those in Ecuador had tried cigarette. In contrast, no more than one in seven women in African or Asian countries had ever tried tobacco [48].

Amos [49] identified several reasons for a rising trend of smoking in some countries: (1) Use of women as a key target group for advertising tobacco; (2) Rapid changes in women’s socio-economic status, meaning that the women who are most likely to start smoking in large numbers are those who are most affluent, well educated and live in urban areas; and (3) Lack of awareness: Because there is a time lag between the widespread increase of smoking and the health effects, even countries with the longest history of female smoking have not yet experienced full impact of smoking on women’s health [49]. However, there is no doubt that smoking among women causes the same detrimental effects as among men [49]. Research has shown that women who smoke: (1) have a 10-times higher risk of heart disease and an increased risk of stroke if they also use oral contraceptives; (2) have a two-fold increased risk of cervical cancer; and (3) have higher rates of dysmenorrhea, miscarriage, premature labor, and low birth-weight babies [49]. Thus it is clear that the current trend of rising smoking in women need to be halted.

A key international response to this detrimental issue is the creation of FCTC in 2005, which is an international public health treaty directed by WHO. FCTC’s major tasks include a comprehensive ban on tobacco advertising; strong health warnings on tobacco packaging that cover at least 30% (and ideally 50%) of the principal display areas within three years; protection from secondhand smoke in indoor workplaces, public places and public transportation; and measures to reduce the smuggling of tobacco products [48]. Moreover, the treaty also requires disclosure and regulations on tobacco product’s ingredients, tobacco sale to minors, treatment for addiction, and tobacco research, and it supports informational exchange among countries to promote awareness [48]. Regardless the many obstacles that the treaty expected to face in LAC, attachment to the treaty was successful and is increasing. Brazil, one of the world’s leading tobacco producers, ratified the FCTC in October 2005, and Bolivia, with an estimate of 1.5 million smokers, attached to the FCTC with no major issues by tobacco private industries [48].

### 2.11. Alcohol

In 2007, a PAHO publication declared that LAC region leads world statistics on deaths, consumption, and drug abuse attributed to alcohol [50]. The most shocking statement revealed that in 2002 there was a death attributed to alcohol every two minutes just in LAC region [50]. When compared to the rest of the world, LAC consumes 40% more alcohol than the world average. The per capita alcohol consumption in LAC is 8.7 liters, while the global per capita alcohol consumption is 6.2 liters [50]. In LAC, children as early as 10 years start drinking alcohol, and alcohol is the favorite drink among the youth [50,51]. Consumption of alcohol among women has increased in recent years. Women who drink are more vulnerable to domestic violence, sexually transmitted diseases including HIV, and sexual abuse than non-drinkers [51].

PAHO recommends the following steps to be taken: establish legislative mechanisms and regulations for the proper production and sale of alcoholic beverages; establish a tax system for alcoholic beverages; regulate or ban advertising or promoting alcoholic beverages; address the issue of drunk driving; develop information systems to monitor the consumption and harms of excessive alcohol consumption; increase public awareness and support for effective policies; and consider alcohol in trade agreements as a special commodity [50]. Moreover, WHO has created the Alcohol and Substance Abuse Program to provide leadership and technical cooperation to its members [52]. A 2008 press release by the WHO/PAHO appreciated Brazil for promulgating a legislation on alcohol and driving, setting therefore an example in the region. With the new law, the government of Brazil has altered prior laws related to road safety and alcohol, setting up a virtual zero level of blood alcohol content while driving [53]. This legislation also creates stronger restrictions on advertising, meaning that all establishments that sell alcohol must place warnings about the danger of drinking and driving.

### 2.12. Indicators of Health

Ever since the epidemiological transition that most countries face, indicators of health have been reshaped and reconfigured. While the leading health indicators in 2010 include physical activity, obesity, mental health, and immunizations, traditional mortality health indicators are still highly valuable for describing health status of a country. Therefore, even though all previous aspects are indicators of health, the following section will focus on major mortality indicators such as IMR and maternal mortality rate (MMR) [54].

**Figure 2.**
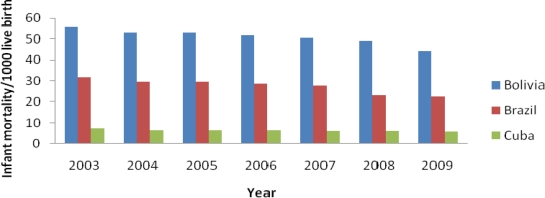
Comparison of infant mortality rates in Bolivia, Brazil and Cuba, 2003–2009. Source: Index Mundi, 2009 [57–59].

*Infant Mortality Rate* (IMR): LAC has been able to maintain a steady decrease of IMR of 4% over a decade since 1990 [10]. Vaccination programs, better access to drinking water, and lower cost for primary care are amongst the principal contributors to decrease IMR [55]. Among the most successful health reforms targeting infants and mothers are the ones implemented by the Brazilian government with a continuity care for women and children; Bolivia’s national health insurance initiatives for women and children; and Ecuador’s free maternity program [56]. Brazil, Chile, Ecuador, Paraguay and Peru offer midwife programs with university certification, and nursing training [56]. Brazil is on track at reducing IMR by 9.16 points from a rate of 31.74 per 1,000 live births in 2003 to 22.58 per 1,000 live births in 2009 (Figure 2), while Bolivia’s IMR is still high and not on track. Cuba stands as the first LAC country that has already fulfilled the MDG by reducing its IMR to 5.82 per 1,000 live births, the lowest rate in the region [55] (Figure 2).

*Maternal mortality rate* (MMR): Since 1980, special international attention has been given to improving maternal health and reducing MMR. Almost 99% of maternal deaths around the globe occur in developing countries [60]. LAC ranks second from the bottom on the rates of MMR in developing nations [60]. The lack of reliable data at the national and regional level forces WHO rely upon mathematical models to assess the burden of MMR [56]. Therefore, to unify data and statistics about MMR, national and regional estimates come from the WHO/UNICEF/UNFPA/World Bank data, which might vary from national estimates of MMR.

For 2005, LAC had 130 maternal deaths per 100,000 live births; and 15,000 total maternal deaths, which shows a significant reduction from the estimates of 1990 [60]. UNICEF reports that Bolivia has one of the highest MMR in the region, with 390 maternal deaths per 100,000 live births from 1990 to 1994, decreasing only to 290 per 100,000 live births in 2005 and to 229 maternal deaths per 100,000 live births in 2009 [60]. The maternal mortality rate in Brazil has dropped from 156 per 100,000 live births to 114 per 100,000 live births; however, it is still 3 to 10 times higher than in other countries with a similar economic status [60]. The Federal Parliamentary Commission of Inquiry on Maternal Mortality found that about 66% of MMR were directly related to Brazil’s inadequate and resource-lacking health system [61]. Similar to Bolivia, the internal discrepancies of MMR within Brazil are marked by socio-economic status and ethnicity, where afro-descendents, mulatto, indigenous, and poor women are the most likely to die from maternal complications.

Since 1980, Cuba has declared health care for women and children as number-one public health priority [60]. According to Cruz, MMR has decreased in Cuba by 78%, from 137.8 per 100,000 live births in 1959 to 21.2 per 100,000 live births in 2005 [62]. Major efforts to control and reduce MMR in Cuba include: continued training of maternity personnel; major emphasis to primary health; and creation and expansion of clinics, hospitals, and health centers in the country [62].

There is a clear link between high rates of MMR and high numbers of illegal abortions [63]. Unsafe abortions, mostly performed by untrained providers and under unhygienic conditions kill an estimated 78,000 women a year or about 13% of all maternal deaths [63]. Attention in reproductive health care, such as the use of contraceptive methods, decreasing fertility, expanding inter-pregnancy intervals, and providing safe abortion care may have great impact in reducing MMR and in improving women’s health.

### 2.13. Prevention of Diseases

Since the burden of disease attributed to chronic conditions is expected to rise, there has been a major shift of attention from infectious disease to chronic disease prevention. PAHO calls for a *Regional Strategy and Plan of Action in Latin America and the Caribbean, 2008–2012* [21]. The major goal of this strategy is based on developing cost-effective approaches in screening and treatment for cervical cancer, among other chronic diseases [21]. Brazil has been identified as a pilot country for chronic disease prevention in the region. Brazil’s behavioral risk factor surveillance by phone, called VIGITEL, was launched in 2006 under direct support of Brazil ministry of health and PAHO. VIGITEL’s main goal involves telephone surveillance to assess demographic and socioeconomic characteristics, dietary patterns, physical activities, body composition, cigarette and alcohol use, self-evaluation of health, and referral for high blood pressure and high cholesterol [21].

## 3. Conclusions

It is crucial to mention that the inconsistency in statistics and data collection methods is among one of the strongest barriers to assess the current health status of LAC. Besides, while clustering all Latin American countries as one region provides some advantages, it also creates enormous issues. In this study, for example, we identified that some LAC countries, such as Cuba, are progressing well and on track, while some other countries within the region have still a long way to fully achieve MDG.

Gender inequalities were identified as a major barrier towards achieving MDG in LAC countries. Gender inequalities exist in term of coverage of health insurance. In some countries in the region, omission of reproductive rights limits the moves towards improving maternal health. In this review, HSR was found to be another barrier. In many LAC countries, HSR has not contributed to the desired goal of reducing inequalities of healthcare access, rather increased the gap in health services between individuals of different income groups.

Considering the changing demographics, it is important to address the future needs of health care for an aging population. While it is important to give attention to the control and prevention of chronic diseases, it is equally important to address the situation of infectious diseases which causes a great majority of childhood deaths in LAC. In Haiti, for example, drug-resistant tuberculosis is a continuing challenge. The double burden of neglected tropical and infectious diseases as well as chronic diseases must be addressed towards reducing health disparities.

Vaccination and immunization are arenas where LAC stands for its tremendous achievements. Even in the poorest countries such as Bolivia, immunization campaigns cover an extensive part of the population. Conversely, measures are needed to improve sanitation, drinking water and nutrition, reduce tobacco and alcohol use, and enhance screening programs in LAC to be on the track to achieve MDG.

## Figures and Tables

**Table 1.** Deaths (thousands) by cause in the Latin America and the Caribbean Region, 2001.

*Population (millions)*	526	
All causes (thousand)	3,277	Rank
*I. Communicable, maternal, perinatal, and nutritional conditions*	716	
Infectious and parasitic diseases	324	3
Respiratory infections	160	8
Perinatal conditions	164	7
*II. Noncommunicable diseases*	2,187	
Malignant neoplasms	484	2
Diabetes mellitus	163	8
Cardiovascular diseases	910	1
Chronic respiratory diseases	195	5
*III. Injuries*	374	
A. Unintentional injuries	207	4
B. Intentional injuries	167	6
